# Distributed lag interrupted time series model for unclear intervention timing: effect of a statement of emergency during COVID-19 pandemic

**DOI:** 10.1186/s12874-022-01662-1

**Published:** 2022-07-25

**Authors:** Daisuke Yoneoka, Takayuki Kawashima, Yuta Tanoue, Shuhei Nomura, Akifumi Eguchi

**Affiliations:** 1 Infectious Disease Surveillance Center, National Institute of Infectious Diseases, Tokyo, Japan; 2 Graduate School of Medicine, The University of Tokyo, Tokyo, Japan; 3 Department of Mathematical and Computing Science, Tokyo Institute of Technology, Tokyo, Japan; 4 Institute for Business and Finance, Waseda University, Tokyo, Japan; 5 School of Medicine, Keio University, Tokyo, Japan; 6 Center for Preventive Medical Sciences, Chiba University, Chiba, Japan

**Keywords:** Interrupted time series, Distributed lag, Unclear intervention timing, Autoregressive integrated moving average model, COVID-19, Human mobility index

## Abstract

**Background:**

Interrupted time series (ITS) analysis has become a popular design to evaluate the effects of health interventions. However, the most common formulation for ITS, the linear segmented regression, is not always adequate, especially when the timing of the intervention is unclear. In this study, we propose a new model to overcome this limitation.

**Methods:**

We propose a new ITS model, *ARIMAITS-DL*, that combines (1) the Autoregressive Integrated Moving Average (ARIMA) model and (2) distributed lag functional terms. The ARIMA technique allows us to model autocorrelation, which is frequently observed in time series data, and the decaying cumulative effect of the intervention. By contrast, the distributed lag functional terms represent the idea that the intervention effect does not start at a fixed time point but is distributed over a certain interval (thus, the intervention timing seems unclear). We discuss how to select the distribution of the effect, the model construction process, diagnosing the model fitting, and interpreting the results. Further, our model is implemented as an example of a statement of emergency (SoE) during the coronavirus disease 2019 pandemic in Japan.

**Results:**

We illustrate the ARIMAITS-DL model with some practical distributed lag terms to examine the effect of the SoE on human mobility in Japan. We confirm that the SoE was successful in reducing the movement of people (15.0–16.0% reduction in Tokyo), at least between February 20 and May 19, 2020. We also provide the R code for other researchers to easily replicate our method.

**Conclusions:**

Our model, ARIMAITS-DL, is a useful tool as it can account for the unclear intervention timing and distributed lag effect with autocorrelation and allows for flexible modeling of different types of impacts such as uniformly or normally distributed impact over time.

**Supplementary Information:**

The online version contains supplementary material available at (10.1186/s12874-022-01662-1).

## Introduction

Interrupted time series (ITS) has become a popular study design to evaluate the effects of health interventions in the field of public health and epidemiology [1, 2]. The effect of the intervention is estimated by comparing it with the “counterfactual" that is estimated by the expected trend assuming the absence of the intervention. ITS has been regarded as one of the best designs to estimate the causality of the intervention, especially when no control population is available, or randomized controlled trials (RCTs) are not feasible [3, 4]. Fretheim et al. (2015) demonstrated that ITS could generate effect estimates similar to those of RCTs [5]. Previous studies extensively examined the strengths and limitations of ITS and provided a practical guideline for its application [3, 6–8].

More formally, let *Y*_*t*_ be an outcome of interest at time *t*, and *T*_0_ be the time of the intervention of interest. For statistical inference, we assume that the sampled time series data $\{Y_{t}\}_{t=1}^{T}$ is available. Standard ITS can be expressed as a simple segmented linear regression formulation, which is given by:

1 $$Y_{t} = \beta_{0} + \beta_{1}t + \beta_{2} D_{t} + \beta_{3} {tD}_{t} + \varepsilon_{t},$$

where *ε*_*t*_ is Gaussian white noise with constant variance $\sigma ^{2}_{\varepsilon }$ (i.e., independent of *t*) and *D*_*t*_ is a dummy variable indicating the post-intervention interval, coded as 0 in the pre-interruption period and 1 in the post-interruption period. *β*_0_ represents the baseline level at *t*=0, *β*_1_ denotes the change in outcome associated with a one-time increase and is regarded as the underlying pre-intervention trend, and *β*_2_ and *β*_3_ represent the changes in the intercept and slope of the trend after the intervention, respectively. Note that although we tentatively use the linear regression formulation expressed in Eq. (1), it can be easily extended to the generalized linear regression formulation.

One issue that has not yet been covered in detail in the prior literature is how the unclear timing of the intervention should be modeled. For example, as we discuss in “Application data analysis” section, consider the case where the statement of emergency (SoE) during the coronavirus disease 2019 (COVID-19) pandemic is the intervention for controlling the spread of infection. Although the date of the SoE declaration is fixed (i.e., *nominal timing* of the intervention is fixed and clear), the actual effect of the SoE begins long before the date of declaration because the media broadcast the declaration in advance, leading to a change in people’s behavior accordingly. In such a case, determining *D*_*t*_ in Eq. (1) becomes difficult as the *actual timing* of the intervention is unclear. In other words, the current ITS approach can only be employed when the timing of the intervention is clear and the nominal timing is the same as the actual timing of the intervention. To overcome this limitation, in this study, we aim to develop a new model that combines the ITS model in Eq. (1) with the 1) autoregressive integrated moving average (ARIMA) model and 2) distributed lag functional terms. The motivation to include the ARIMA technique is 1-A) to model autocorrelation, which is frequently observed in time series data but cannot be treated well in Eq. (1), and 1-B) to model the decaying cumulative effect of the intervention. By contrast, the motivation to include the distributed lag functional terms is 2-A) to represent the idea that the effect of the intervention does not start at a fixed time point but that the intervention timing is distributed over a certain interval (thus, the intervention timing is unclear), and 2-B) to model the distributed effect of the intervention over a certain period that includes the timing of the intervention.

The remainder of this study is structured as follows. In “Methods” section, we introduce the idea of the ARIMA ITS model with distributed lag functional terms to model the unclear intervention timing and its distributed effect and then describe the inference procedure. In “Application data analysis” section, we outline practical data analysis procedures of our method for the users by applying several human mobility datasets during the COVID-19 pandemic in Japan. In “Discussion” section, we discuss and propose possible further developments.

## Methods

### ARIMAX iTS with distributed lag functional terms

In this section, we first explain an ARIMA model with exogenous variables (ARIMAX) [9]. Then, by extending the exogenous variables to our new functional variables (*distributed lag functional terms*), we propose our model, *ARIMAITS-DL*, to simultaneously model both the unclear intervention timing and the distributed effect of the intervention.

Define the sampled time series data as $\{Y_{t}, \boldsymbol {X}_{t}\}_{t=1}^{T}$. The general class of the ARIMAX (*p,d,q*) model without a constant takes the form of; [9, 10]

2 $$\delta(B)\Delta^{d} Y_{t} = \boldsymbol{X}_{t}\boldsymbol{\beta} + \theta(B)\varepsilon_{t},$$

or equivalently,

3 $$\Delta^{d}Y_{t} = \delta(B)^{-1}\boldsymbol{X}_{t}\boldsymbol{\beta} + \frac{\theta(B)}{\delta(B)}\varepsilon_{t},$$

where *B* is a lag operator such that ${BY}_{t} = Y_{t-1},\ \delta (B) = 1 - \delta _ {1} B -\dots -\delta_{p}B^{p}=1-\sum_{i=1}^{p}\delta_{i}B^{i},\ \delta_{i}<1,\ \Delta = (1-B),\ \theta (B) = 1 - \theta_ {1} B -\dots -\theta_{q}B^{q}=1-\sum_{i=1}^{q}\theta_{i}B^{i},\ \delta_{i}$ is the parameter for the autoregressive (AR) part, *θ*_*i*_ is the parameter for the moving average part (MA), and *p* and *q* are the orders of the AR and MA parts, respectively. For simplicity, we set *d*=0, and thus, ARIMAX(*p*,*d*,*q*) reduces to ARMAX (*p,q*). Even when the model where *d*>0 is needed, the following procedure is still valid after differencing *Y*_*t*_ before fitting the model. The example of an analytical procedure in this case is explained in “Application data analysis” section. When the covariate vector ***X*** includes a dummy variable indicating the post-intervention intervals, which corresponds to *D*_*t*_ in Eq. (1), we can extend the simple ITS model to the ARIMA model [11].

To model both the unclear intervention timing and the distributed effect of the intervention over time, we propose the following ARIMAX ITS model with distributed lag functional terms with the covariates ***X***_*t*_, denoted by ARIMAITS-DL (*p*,*q*,*l*_1_,*l*_2_). We define *T*_0_ as the nominal timing of the intervention.

4 $$(1-\sum\limits_{i=1}^{p}\delta_{i}B^{i})Y_{t} = \sum\limits_{k=0}^{l_{1}+l_{2}}w_{k}F_{t-k}+\boldsymbol{X_{t}\beta}+(1-\sum\limits_{i=1}^{q}\theta_{i}B^{i})\varepsilon_{t},$$

where $\{\delta _{i}\}_{i=1}^{p}, \{w_{k}\}_{k=0}^{l_{1}+l_{2}}, \boldsymbol {\beta }^{T}$, and $\{\theta _{i}\}_{i=1}^{q}$ are unknown parameters,

5 $$F_{t-k} = \left\{\begin{array}{ll} f(t-k-T_{0}+l_{1}) \ \ & \text{if}\ t\ \in [T_{0}-l_{1}+k, T_{0}+l_{2}] \\ 0 \ \ & \text{Otherwise}, \end{array}\right.$$

*l*_1_ controls the duration before the effect of the intervention appears (i.e., the start timing of the effect), and *l*_2_ controls the duration of the effect (i.e., the end timing of the effect). Thus, the effect of the intervention is assumed to last from *T*_0_−*l*_1_ to *T*_0_+*l*_2_. Given this formalization, we can model the unclear intervention timing during *t*∈[*T*_0_−*l*_1_,*T*_0_+*l*_2_]. The distributed lag functional term *F*_*t*−*k*_ represents how the effect of the intervention is distributed over the time during *t*∈[*T*_0_−*l*_1_+*k,T*_0_+*l*_2_]. The function *f*() is a probability density function (pdf) of time *t* that represents the proportion of the distributed effect of the intervention. Note that Eq. (4) can be considered one special case of the transfer function model popularized by Box and Jenkins [10].

**Fig. 1:**
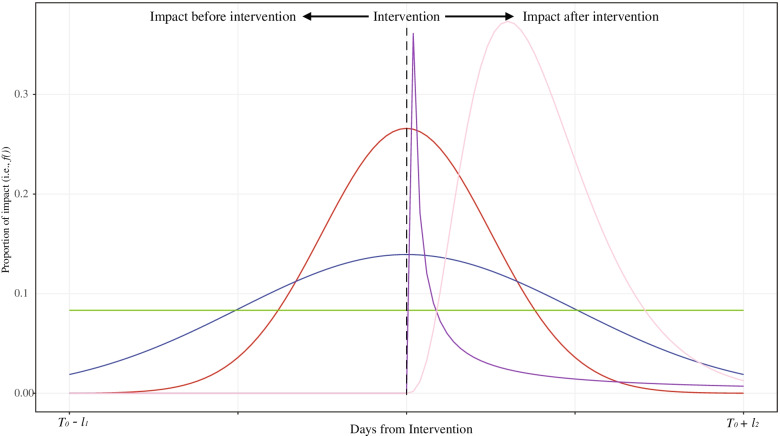
Examples of *f*() corresponding to the proportion of the effect of intervention at time of *T*_0_ between *t*∈[*T*_0_−*l*_1_,*T*_0_+*l*_2_]

Figure 1 illustrates examples of *f*(): ex. if the effect is assumed to be distributed uniformly and symmetrically with a peak at *T*_0_ or asymmetrically around *T*_0_, *f*() might be formulated as a uniform distribution (green line in Fig. 1), (truncated) normal distribution (blue or red lines in Fig. 1), or (truncated) log-normal distribution (purple in Fig. 1), respectively. The choice of *f*() is discussed in the following section with practical examples.

### Detailed explanation on distributed lag functional terms

For simplicity, we set *p*=1 and *q*=0 and drop ***X***_*t*_ from Eq. (4). Thus, our model reduces to the ARX(1,0) model with distributed lag functional terms. Then, Eq. (4) is the reduced simple form that can be expressed as:

6 $$Y_{t} = \delta Y_{t-1} + \sum\limits_{k=0}^{l_{1}+l_{2}}w_{k}F_{t-k}+\varepsilon_{t},$$

where |*δ*|<1 is assumed to make *Y*_*t*_ a stationary process. Now, *t* is assumed to indicate a day. *F*_*t*−*k*_ indicates the proportion of the distributed effect of the intervention and *w*_*k*_ estimates the (lagged) effect. Thus, the term *w*_*k*_*F*_*t*−*k*_ indicates the distributed lag effect on *Y*_*t*_. Another advantage of the formulation of Eq. (6) is that because of the AR parameter, *δ*, we can model the cumulative effect of the intervention from the previous time point. For a clearer understanding, we explain Eq. (6) with examples as follows.

From the definition of *F*_*t*−*k*_, when *t*<*T*_0_−*l*_1_, Eq. (6) becomes

7 $$Y_{t} = \delta Y_{t-1}+\varepsilon_{t}.$$

Consider the case where *t*∈[*T*_0_−*l*_1_,*T*_0_+*l*_2_] (i.e., during the intervention period). When *t*=*T*_0_−*l*_1_+*m* (*m*∈[0,*l*_1_+*l*_2_]), the iterative use of Eq. (6) results in the general form as

8 $$Y_{t} = \delta^{m-1}Y_{T_{0}-l_{1}-1} + \sum\limits_{j=0}^{m}\sum\limits_{i=0}^{m-j}\delta^{m-j-i}w_{i}F_{T_{0}-l_{1}+j}+\sum\limits_{i=0}^{m}\delta^{m-i}\varepsilon_{T_{0}-l_{1}+i}.$$

This formulation represents the cumulative effect of the intervention since a day before the intervention becomes effective (i.e., the day of *T*_0_−*l*_1_−1). The first term is the cumulative effect of the outcome $Y_{T_{0}-l_{1}-1}$ with decay *δ*^*k*−1^, the second term is the delayed and cumulative effect of the intervention, which is explained below, and the third term is an error term.

Finally, we consider the post-intervention period (i.e., *t*>*T*_0_+*l*_2_). When *t*>*T*_0_+*l*_2_+*n* (*n*∈[0,*l*_1_+*l*_2_]), Eq. (6) becomes

9 $$Y_{t} = \delta Y_{t-1} + \sum\limits_{k=n}^{l_{1}+l_{2}}w_{k}F_{t-k}+\varepsilon_{t},$$

and when *t*>*T*_0_+*l*_1_+2*l*_2_, Eq. (6) becomes the following simple form again as:

10 $$Y_{t} = \delta Y_{t-1} + \varepsilon_{t}.$$

Now by using Eq. (6) iteratively, we obtain the general form Eq. (8). For a clearer understanding, we introduce the following example calculations, (T0-T2), which correspond to the first three days after the actual intervention becomes effective (i.e., the *actual* timing of the intervention while the *nominal* intervention timing is still *T*_0_). T0. When *t*=*T*_0_−*l*_1_, the outcome $Y_{T_{0}-l_{1}}$ is affected by the white noise $\varepsilon _{T_{0}-l_{1}}$, the decayed outcome at one day ago $\delta Y_{T_{0}-l_{1}-1}$, where |*δ*|<1 represents the decay rate and the proportion of the effect of intervention at the first date $F_{T_{0}-l_{1}}=f(0)$, and *w*_0_, which represents the effect size as follows:

$$Y_{T_{0}-l_{1}} = \delta Y_{T_{0}-l_{1}-1} + \underbrace{w_{0}F_{T_{0}-l_{1}}}_{\substack{\text{effect of 0 day} \\ w_{0}:\ \text{0-day-delayed effect} \\ F_{T_{0}-l_{1}}: \%\text{ of effect at 0 day}}} + \varepsilon_{T_{0}-l_{1}}.$$

T1. When *t*=*T*_0_−*l*_1_+1, the outcome $Y_{T_{0}-l_{1}+1}$ is affected by the white noise $\varepsilon _{T_{0}-l_{1}+1}$, the decayed outcome at $\delta Y_{T_{0}-l_{1}}$, the proportion of the effect of intervention on the first and second dates $F_{T_{0}-l_{1}+1} =f(1), F_{T_{0}-l_{1}}=f(0)$, and *w*_0_ and *w*_1_, which represent the 0- or 1-day delayed effect), respectively, as follows:

$$\begin{array}{*{20}l} Y_{T_{0}-l_{1}+1} &= \delta Y_{T_{0}-l_{1}} + \underbrace{w_{0}F_{T_{0}-l_{1}+1}}_{\substack{\text{effect of}\ +1 \text{day} \\ w_{0}:\ \text{0-day-delayed effect} \\ F_{T_{0}-l_{1}+1}: \%\text{ of effect at \(+1\) day}}}+\underbrace{w_{1}F_{T_{0}-l_{1}}}_{\substack{\text{effect of 0 day}\\ w_{1}:\ \text{1-day-delayed effect}\\ F_{T_{0}-l_{1}}: \%\text{ of effect at 0 day}}} + \varepsilon_{T_{0}-l_{1}+1}. \end{array}$$

By plugging $Y_{T_{0}-l_{1}}$ in T0, we can obtain the following equation:

$$\begin{array}{*{20}l} Y_{T_{0}-l_{1}+1}&=\delta (\delta Y_{T_{0}-l_{1}-1} + w_{0}F_{T_{0}-l_{1}}+\varepsilon_{T_{0}-l_{1}})+w_{0}F_{T_{0}-l_{1}+1}\\ & +w_{1}F_{T_{0}-l_{1}}+\varepsilon_{T_{0}-l_{1}+1}\\ &= \delta^{2}Y_{T_{0}-l_{1}-1} + \underbrace{w_{0}F_{T_{0}-l_{1}+1}}_{\text{effect of}\ +1 \text{day}}+ \underbrace{\underbrace{(\delta w_{0}+w_{1})}_{\substack{\delta w_{0}:\ \text{0-day-delayed effect with decay}\ \delta \\ w_{1}:\ \text{1-day-delayed effect}}}}_{\text{effect of 0 day}}\!\!\!\!\!\!\!\!\!\!\!\!\!\!\!\!\! F_{T_{0}-l_{1}}\\ &+\varepsilon_{T_{0}-l_{1}+1}+\delta\varepsilon_{T_{0}-l_{1}} \end{array}$$

T2. When *t*=*T*_0_−*l*_1_+2, we obtain $F_{T_{0}-l_{1}+2} =f(2), F_{T_{0}-l_{1}+1}=f(1), F_{T_{0}-l_{1}}=f(0)$. Using Eq. (6) iteratively, the model is reduced to:

11 $$\begin{array}{*{20}l} Y_{T_{0}-l_{1}+2} &= \delta Y_{T_{0}-l_{1}+1} + \underbrace{w_{0}F_{T_{0}-l_{1}+2}}_{\substack{\text{effect of}\ +2\ \text{day} \\ w_{0}:\ \text{0-day-delayed effect} \\ F_{T_{0}-l_{1}+2}:\ \%\text{ of effect at \(+2\) day}}}+ \underbrace{w_{1}F_{T_{0}-l_{1}+1}}_{\substack{\text{effect of}\ +1 \text{day} \\ w_{1}:\ \text{1-day-delayed effect} \\ F_{T_{0}-l_{1}+1}:\ \%\text{ of effect at}\ +1 \text{day}}} \\ &+\underbrace{w_{2}F_{T_{0}-l_{1}}}_{\substack{\text{effect of 0 day} \\ w_{2}:\ \text{2-day-delayed effect} \\ F_{T_{0}-l_{1}}:\ \%\text{ of effect at 0 day}}}+\varepsilon_{T_{0}-l_{1}+1} \\ &=\delta^{3} Y_{T_{0}-l_{1}-1}+\underbrace{w_{0}F_{T_{0}-l_{1}+2}}_{\text{effect of}\ +2 \text{day}}+\underbrace{\underbrace{(\delta w_{0}+w_{1})}_{\substack{\delta w_{0}:\ \text{0-day-delayed effect with decay}\ \delta \\ w_{1}:\ \text{1-day-delayed effect}}}F_{T_{0}-l_{1}+1}}_{\text{effect of}\ +1 \text{day}} \\ &+ \underbrace{\underbrace{(\delta^{2}w_{0}+\delta w_{1}+w_{2})}_{\substack{\delta^{2} w_{0}:\ \text{0-day-delayed effect with decay}\ \delta^{2} \\ \delta w_{1}:\ \text{1-day-delayed effect with decay}\ \delta \\ w_{2}:\ \text{2-day-delayed effect}}}F_{T_{0}-l_{1}+1}}_{\text{effect of 0 day}} \\ &+\varepsilon_{T_{0}-l_{1}+2}+\delta\varepsilon_{T_{0}-l_{1}+1}+\delta^{2}\varepsilon_{T_{0}-l_{1}}. \end{array}$$

For example, Eq. (11) provides an intuitive explanation of the cumulative and delayed effect of the intervention. The first term is the decayed outcome at *T*_0_−*l*_1_−1; the second term is the effect on the same day (i.e., *T*_0_−*l*_1_+2), which is decomposed into the 0-day-delayed effect *w*_0_ and the proportion of effect on the same day $F_{T_{0}-l_{1}+2} =f(2)$; the third term is the cumulative and delayed effect from the previous day, which is decomposed into the 1-day-delayed effect *w*_1_, the decayed 0-day-delayed effect *δw*_0_, and the proportion of effect of the previous day $F_{T_{0}-l_{1}+1} =f(1)$; and the fourth term is the cumulative and delayed effect from 2-day ago, which is decomposed into the 2-day-delayed effect *w*_2_, the decayed 0- and 1-day-delayed effects *δ*^2^*w*_0_,*δw*_1_, and the proportion of effect from 2-day ago $F_{T_{0}-l_{1}} =f(0)$. This example explains that we can model the proportion of the intervention on a given day and the cumulative and delayed effects of the intervention from the previous days.

### Restrictions for estimation

The simplest procedure for estimating the parameters in Eq. (4) is using the maximum likelihood method as with the ordinary ARIMA modeling. Unfortunately, the precision of the estimates of $\{w_{k}\}_{k=0}^{l_{1}+l_{2}}$ is known to sometimes become poor because of the high correlation among $\{F_{t-k}\}_{k=0}^{l_{1}+l_{2}}$, resulting in multicollinearity in the model [12, 13]. To obtain stable estimates of *w*_*k*_s, we impose the following constraints: We redefine $\sum_{k=0}^{l_{1}+l_{2}}w_{k}F_{t-k}$ in a matrix form as:

12 $$\begin{array}{*{20}l} \sum\limits_{k=0}^{l_{1}+l_{2}}w_{k}F_{t-k} = \boldsymbol{F}^{T}\boldsymbol{C\eta}, \end{array}$$

where $\boldsymbol {F}=(F_{t},\dots,F_{t-l_{1}-l_{2}})^{T}\in \mathbb {R}^{l_{1}+l_{2}+1},\ \boldsymbol {C\eta }=(w_{0},\dots,w_{l_{1}+l_{2}})^{T}\in \mathbb {R}^{l_{1}+l_{2}+1},\ \boldsymbol {C}\in \mathbb {R}^{(l_{1}+l_{2}+1)\times h}$ includes the basis variables derived from the specific constraint on *w*_*k*_s, and $\boldsymbol {\eta }\in \mathbb {R}^{h}$ is a vector of unknown parameters. For example, a constant decline during the lag interval is modeled by:

13 $$\begin{array}{*{20}l} \boldsymbol{C\eta} = \left(\begin{array}{c} \frac{l_{1}+l_{2}}{l_{1}+l_{2}+1} \\ \vdots \\ \frac{1}{l_{1}+l_{2}+1} \end{array}\right) \eta_{0}, \end{array}$$

a moving average in the previous *L* period is modeled by (in the case of *L*=2):

14 $$\begin{array}{*{20}l} \boldsymbol{C\eta} = \left(\begin{array}{cc} 1 & 0 \\ 0 & 1 \\ 1/2 & 1/2 \\ 1/4 & 3/4\\ \vdots & \vdots \\ \cfrac{2^{l_{1}+l_{2}-1}-(-1)^{l_{1}+l_{2}-1}}{3\times 2^{l_{1}+l_{2}-1}} & 1-\cfrac{2^{l_{1}+l_{2}-1}-(-1)^{l_{1}+l_{2}-1}}{3\times 2^{l_{1}+l_{2}-1}} \end{array}\right) \left(\begin{array}{cc} \eta_{0} \\ \eta_{1} \end{array}\right), \end{array}$$

and a polynomial smoothing proposed by Schwartz (2000) [11] and Rondeau et al. (2004) [14] is modeled by:

15 $$\begin{array}{*{20}l} \boldsymbol{C\eta} = \left(\begin{array}{cccccc} 1 & 0 & 0 & \dots & 0 & 0 \\ 1 & 1 & 1 & \dots & 1 & 1 \\ 1 & 2 & 2^{2} & \dots & 2^{s-1} & 2^{s} \\ & & \ddots \\ 1 & (l_{1}+l_{2}) & (l_{1}+l_{2})^{2} & \dots & (l_{1}+l_{2})^{s-1} & (l_{1}+l_{2})^{s} \end{array}\right) \left(\begin{array}{c} \eta_{0} \\ \eta_{1} \\ \vdots \\ \eta_{s} \end{array}\right), \end{array}$$

where *s* denotes the degree of the polynomial function. Other examples with non-linear functions, such as splines, can be found in Gasparrini et al. (2010) [13]. These models have been popular for modeling air pollution [15] and temperatures [16].

### Modeling steps, the selection of orders (*p,q,l*_1_,*l*_2_) and *f*(), and creating counterfactual

We follow the Box–Jenkins three-stage modeling strategy, including identification, estimation, and diagnostic checking [10]. At the identification stage, a visual inspection of the plots for the time series data allows us to check some important features, such as structural changes, outliers, and missing values, and ensure the stationarity of the time series data. Non-stationary time series invalidates the analyses with the ordinary ARIMA model. To check the stationarity, several tests, such as the Augmented Dickey–Fuller (ADF) test, Ljung–Box test, and Kwiatkowski–Philips–Schmidt–Shin test, can be used [9]. If the time series is judged as non-stationary, a common approach is to subtract successive observations—also known as differencing—to stationarize the time series data. By iteratively applying this test-differencing approach, we can select the order *d* in Eq. (4). Once *d* is fixed, *Y*_*t*_ in Eq. (4) can be replaced with the differentiated *Y*_*t*_.

The plots of the autocorrelation function (ACF) and partial autocorrelation function (PACF) should be visually examined to identify the search range of the orders of the AR and MA parameters: 1) to decide an AR(*p*) model, the ACF should slowly decrease and PACF should cut off after lag *p*; 2) to decide an MA(*q*) model, the PACF should slowly decline and the ACF should cut off after lag *q*; and 3) to decide an ARMA(*p*,*q*) model, both the ACF and PACF should tail off.

At the estimation stage, the maximum likelihood method is used to estimate the regression parameters. The Akaike information criterion (AIC) or Bayesian information criterion (BIC) can be used to select the optimal order of (*p*,*q*) among the aforementioned search range defined by the ACF or PACF. In addition, regarding the search space defined by the possible combinations of (*l*_1_,*l*_2_) and *f*(), they can be specified by the researcher based on the literature search or expert opinion; however, the sensitivity analysis for their choice or model fitting statistics such as AIC or BIC can be helpful. In “Application data analysis” section, we check the sensitivity of the results by changing the combination of (*l*_1_,*l*_2_) and *f*(). The last step is to check the residuals of the selected model by visual inspection of the residual plot and by testing the presence of autocorrelation using methods such as the Ljung–Box test for white noise. If the autocorrelation is still judged to exist in the residuals, different AR and/or MA orders can be chosen.

Finally, once the regression parameters are estimated, the intervention effect can be estimated by calculating the differences between the observed data and counterfactual prediction. The counterfactual (i.e., *Y*_*t*_ in the absence of the intervention) can be created by substituting 0 into all the components in *F*_*t*−*k*_s.

## Application data analysis

### Data description

In response to the global COVID-19 pandemic, governments implemented large-scale public health interventions, such as lockdowns and SoE declarations, to control the spread of the virus. To quantify the effect of such interventions, human mobility has been frequently measured in terms of tracing human contact in several places, such as restaurants and workplaces. Previous studies have illustrated that the SoE declaration can effectively reduce human mobility [17–19]. In this study, we use the human mobility index (HMI) in Japan published by Google [20]. The HMI represents the relative percentage changes in the daily number of visitors (or time spent) from the baseline period (i.e., the same day of the 5-week period between January 3 and February 6, 2020) at six locations: retail and recreational places, grocery and pharmacy stores, public transportation (transit) stations, parks and outdoor spaces, workplaces, and residential areas. The HMI is available daily for all 47 prefectures in Japan. For a simple explanation of our method, we mainly use the HMI at workplaces in Tokyo from February 20 to May 19, 2020. In Tokyo, the first SoE was officially declared on April 7, 2020, although the media broadcast the event even before it was declared, which may have impacted human mobility [21, 22]. In this sense, the *nominal* intervention timing is clear, although the *actual* intervention (i.e., the first SoE) timing is unclear. In this section, we demonstrate that the proposed method can handle such cases. Lastly, we use the following covariates in ***X***: the daily COVID-19 test-positive rate, daily number of deaths, and daily (average) temperature.

### Practical procedure of data analysis

**Fig. 2:**
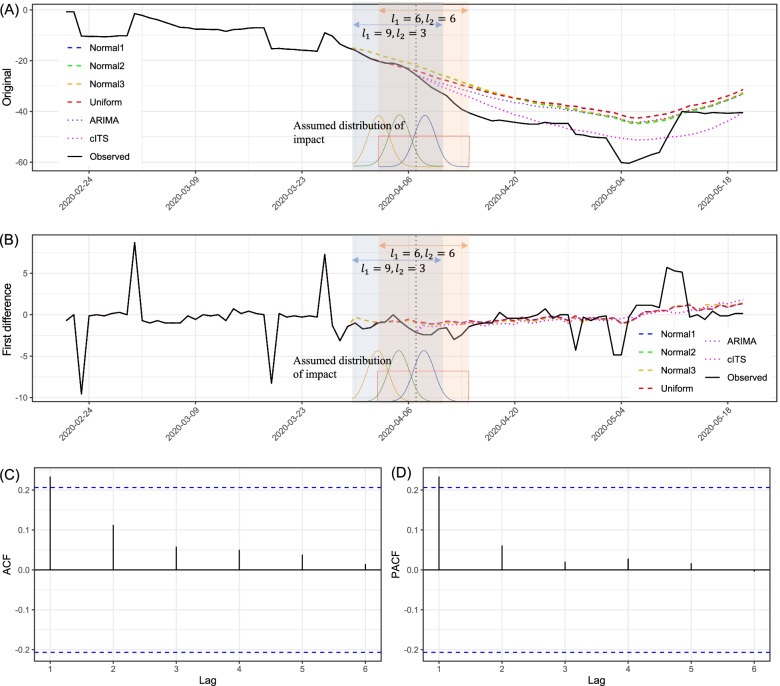
Application results in Tokyo: **A** Original time series, **B** First difference of the data, **C** Autocorrelation function (ACF) plot for the first difference of the data, and **D** Partial autocorrelation function (PACF) plot for the first difference of the data

The data are illustrated in Fig. 2A, where the observed HMI is plotted as a black solid line. To reduce the variation in HMI by day of the week, a seven-day rolling average is calculated in advance. The ADF test indicates that the original time series is non-stationary (*p*=0.571), and thus, the first difference of the time series data (Fig. 2B) is used to induce stationarity (the ADF test shows *p*=0.049). Therefore, *Y*_*t*_ is the first difference data, and *d*=0 is fixed. The ACF and PACF of the stationary (i.e., first-differenced) data are plotted in Fig. 2C and D, respectively. In the figures, the black solid bars above or below the blue dashed lines represent statistically significant autocorrelation with *p*<0.05. In Fig. 2C and D, we can check that autocorrelation does not exist after lag 1. This implies that the search range of the order for *p* and *q* might be around lag 1 [11]. We then search over a series of potential models for the best model with the lowest AIC by using the *auto.arima()* function in the *forecast* package in *R*. Each model is optimized using the maximum likelihood method.

To estimate the effect of the SoE on the HMI in Tokyo, the intervention effect is assumed to be distributed uniformly or normally over time, that is, *f*() in Eq. (5) is set to the pdf of uniform distribution or (truncated) normal distribution (truncated by *l*_1_ and *l*_2_). In addition, for the constraint on the estimation of *w*_*k*_s, both Eqs. (14) and (15) are used. Consequently, we have four types of ITS models by combining two *f*() and two constraints, as follows:

1. *f*() is a normal distribution *N*(0,1) that is truncated by *l*_1_=6 and *l*_2_=6 (i.e., the center is the day of the SoE (April 7, 2020) and truncated at April 1 and 13, 2020). In addition, the constraint for *w*_*k*_s is the polynomial smoothing model Eq. (15), with *s*=3.
2. Similar to Normal1, but *f*() is truncated by different days, that is, *f*() is a normal distribution *N*(0,1) that is truncated by *l*_1_=9 and *l*_2_=3 (i.e., the center is the day of the SoE (April 7, 2020) and truncated at March 29 and April 10, 2020). Moreover, the constraint for *w*_*k*_s is the polynomial smoothing model, Eq. (15), with *s*=3.
3. Similar to Normal2, but *f*() has a different normal distribution, that is, *f*() is a normal distribution *N*(−3,3) that is truncated by *l*_1_=9 and *l*_2_=3 (i.e., the center is 3 days before the SoE (April 4, 2020) and truncated at March 29 and April 10, 2020). Further, the constraint for *w*_*k*_s is the MA model, Eq. (14).
4. *f*() is a uniform distribution with *l*_1_=6 and *l*_2_=6 (i.e., the center is the day of the SoE (April 7, 2020) and truncated at April 1 and 13, 2020). In addition, the constraint for *w*_*k*_s is the MA model, Eq. (14).

The model with the lowest AIC selected by the algorithm is ARIMA(1,0,0) for all the above models. The residuals for all the models have no significant autocorrelation: all *p*-values of the Ljung–Box test for white noise are more than 0.9 at six lags. As the data comprise the first difference (Fig 2B), we calculate it back into the original time series (Fig. 2A). To compare our method with the other approaches, the conventional ITS model (denoted by cITS in Figures and Tables) and the model proposed by Schaffer et al. (2021) [11], which is an ARIMA-based ITS model (denoted by ARIMA in Figures and Tables), are included.

**Fig. 3:**
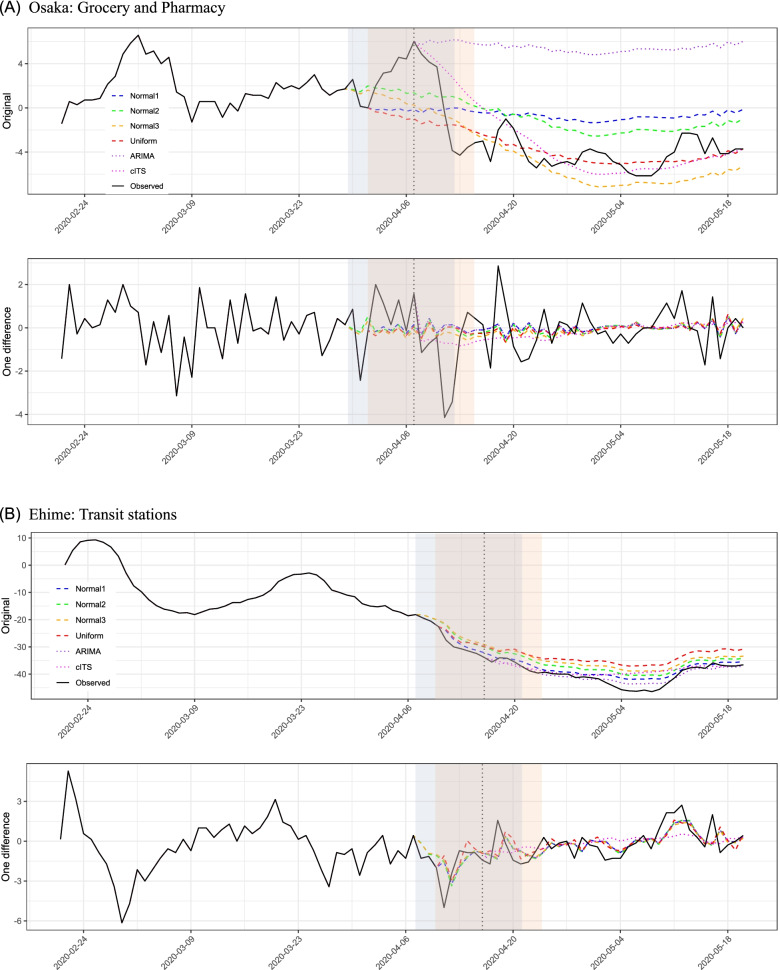
Application results in Osaka and Ehime: **A** Upper: Original time series, Lower: First difference of the data in Osaka **B** Upper: Original time series, Lower: First difference of the data in Ehime

Lastly, we validate our method in different COVID-19 datasets by using different prefectures (Osaka and Ehime prefectures: Osaka is a populous and large prefecture and Ehime is a local and small prefecture in Japan), different HMI at “grocery and pharmacy store” and “public transportation (transit) stations”, and different timing of SoE (Osaka at April 7 and Ehime at April 16, 2020). The same procedures described above are used. Figure 3 and the Additional files include the detailed results of these validations.

### Application results

Figures 2A and B (colored and dashed lines) indicate the observed data and counterfactual predictions in Tokyo by our ARIMAITS-DL models, assuming the absence of the intervention. Dotted lines indicate the comparison groups: cITS (i.e., the conventional ITS model) and ARIMA (i.e., the model by Schaffer et al. (2021)). Table 1 provides more detailed values of the observed and estimated HMIs. It illustrates that the SoE on April 7, 2020, was successful in reducing the mobility of people: Normal1, Normal2, Normal3, and Uniform models show -392.42 (16.0%), -372.33 (15.0%), -388.61 (16.0%), and -392.20 (16.0%) (cumulative) reduction in HMI, respectively, after the SoE became effective in Tokyo. The difference between the observed and counterfactual peaks around the head of May 2020 for all models, Normal1, Normal2, Normal3, and Uniform models, show a reduction of -18.68 (31.1%), -16.66 (27.7%), -17.12 (28.5%), and -18.74 (31.2%), respectively, on May 3, 2020, which corresponds to the middle period of Japan’s long holiday season. In the comparison groups, the similar tendency are observed, while the degree of reduction in HMI are likely to be underestimated: cITS and ARIMA models show -60.15 (3.0%) and -319.21 (15.0%) (cumulative) reduction in HMI, respectively, after the SoE became effective in Tokyo. Importantly, our findings should only be valid for the study period (i.e., February 20 to May 19, 2020, in Tokyo).

**Table 1:** Observed and estimated human mobility index (HMI) at workplaces in Tokyo

Date	First difference							Original						
	Observed	Normal1	Normal2	Normal3	Uniform	cITS	ARIMA	Observed	Normal1	Normal2	Normal3	Uniform	cITS	ARIMA
20-Feb	-0.71	-0.71	-0.71	-0.71	-0.71	-0.71	-0.71	-0.71	-0.71	-0.71	-0.71	-0.71	-0.71	-0.71
21-Feb	0.00	0.00	0.00	0.00	0.00	0.00	0.00	-0.71	-0.71	-0.71	-0.71	-0.71	-0.71	-0.71
22-Feb	-9.57	-9.57	-9.57	-9.57	-9.57	-9.57	-9.57	-10.29	-10.29	-10.29	-10.29	-10.29	-10.29	-10.29
23-Feb	-0.14	-0.14	-0.14	-0.14	-0.14	-0.14	-0.14	-10.43	-10.43	-10.43	-10.43	-10.43	-10.43	-10.43
24-Feb	0.00	0.00	0.00	0.00	0.00	0.00	0.00	-10.43	-10.43	-10.43	-10.43	-10.43	-10.43	-10.43
25-Feb	-0.14	-0.14	-0.14	-0.14	-0.14	-0.14	-0.14	-10.57	-10.57	-10.57	-10.57	-10.57	-10.57	-10.57
26-Feb	0.14	0.14	0.14	0.14	0.14	0.14	0.14	-10.43	-10.43	-10.43	-10.43	-10.43	-10.43	-10.43
27-Feb	0.29	0.29	0.29	0.29	0.29	0.29	0.29	-10.14	-10.14	-10.14	-10.14	-10.14	-10.14	-10.14
28-Feb	0.00	0.00	0.00	0.00	0.00	0.00	0.00	-10.14	-10.14	-10.14	-10.14	-10.14	-10.14	-10.14
29-Feb	8.71	8.71	8.71	8.71	8.71	8.71	8.71	-1.43	-1.43	-1.43	-1.43	-1.43	-1.43	-1.43
01-Mar	-0.71	-0.71	-0.71	-0.71	-0.71	-0.71	-0.71	-2.14	-2.14	-2.14	-2.14	-2.14	-2.14	-2.14
02-Mar	-1.00	-1.00	-1.00	-1.00	-1.00	-1.00	-1.00	-3.14	-3.14	-3.14	-3.14	-3.14	-3.14	-3.14
03-Mar	-0.71	-0.71	-0.71	-0.71	-0.71	-0.71	-0.71	-3.86	-3.86	-3.86	-3.86	-3.86	-3.86	-3.86
04-Mar	-1.00	-1.00	-1.00	-1.00	-1.00	-1.00	-1.00	-4.86	-4.86	-4.86	-4.86	-4.86	-4.86	-4.86
05-Mar	-1.00	-1.00	-1.00	-1.00	-1.00	-1.00	-1.00	-5.86	-5.86	-5.86	-5.86	-5.86	-5.86	-5.86
06-Mar	-1.00	-1.00	-1.00	-1.00	-1.00	-1.00	-1.00	-6.86	-6.86	-6.86	-6.86	-6.86	-6.86	-6.86
07-Mar	-0.14	-0.14	-0.14	-0.14	-0.14	-0.14	-0.14	-7.00	-7.00	-7.00	-7.00	-7.00	-7.00	-7.00
08-Mar	-0.57	-0.57	-0.57	-0.57	-0.57	-0.57	-0.57	-7.57	-7.57	-7.57	-7.57	-7.57	-7.57	-7.57
09-Mar	0.00	0.00	0.00	0.00	0.00	0.00	0.00	-7.57	-7.57	-7.57	-7.57	-7.57	-7.57	-7.57
10-Mar	-0.14	-0.14	-0.14	-0.14	-0.14	-0.14	-0.14	-7.71	-7.71	-7.71	-7.71	-7.71	-7.71	-7.71
11-Mar	0.00	0.00	0.00	0.00	0.00	0.00	0.00	-7.71	-7.71	-7.71	-7.71	-7.71	-7.71	-7.71
12-Mar	-0.71	-0.71	-0.71	-0.71	-0.71	-0.71	-0.71	-8.43	-8.43	-8.43	-8.43	-8.43	-8.43	-8.43
13-Mar	0.71	0.71	0.71	0.71	0.71	0.71	0.71	-7.71	-7.71	-7.71	-7.71	-7.71	-7.71	-7.71
14-Mar	0.14	0.14	0.14	0.14	0.14	0.14	0.14	-7.57	-7.57	-7.57	-7.57	-7.57	-7.57	-7.57
15-Mar	0.43	0.43	0.43	0.43	0.43	0.43	0.43	-7.14	-7.14	-7.14	-7.14	-7.14	-7.14	-7.14
16-Mar	0.14	0.14	0.14	0.14	0.14	0.14	0.14	-7.00	-7.00	-7.00	-7.00	-7.00	-7.00	-7.00
17-Mar	0.00	0.00	0.00	0.00	0.00	0.00	0.00	-7.00	-7.00	-7.00	-7.00	-7.00	-7.00	-7.00
18-Mar	-8.29	-8.29	-8.29	-8.29	-8.29	-8.29	-8.29	-15.29	-15.29	-15.29	-15.29	-15.29	-15.29	-15.29
19-Mar	0.14	0.14	0.14	0.14	0.14	0.14	0.14	-15.14	-15.14	-15.14	-15.14	-15.14	-15.14	-15.14
20-Mar	-0.29	-0.29	-0.29	-0.29	-0.29	-0.29	-0.29	-15.43	-15.43	-15.43	-15.43	-15.43	-15.43	-15.43
21-Mar	-0.14	-0.14	-0.14	-0.14	-0.14	-0.14	-0.14	-15.57	-15.57	-15.57	-15.57	-15.57	-15.57	-15.57
22-Mar	-0.29	-0.29	-0.29	-0.29	-0.29	-0.29	-0.29	-15.86	-15.86	-15.86	-15.86	-15.86	-15.86	-15.86
23-Mar	-0.14	-0.14	-0.14	-0.14	-0.14	-0.14	-0.14	-16.00	-16.00	-16.00	-16.00	-16.00	-16.00	-16.00
24-Mar	-0.29	-0.29	-0.29	-0.29	-0.29	-0.29	-0.29	-16.29	-16.29	-16.29	-16.29	-16.29	-16.29	-16.29
25-Mar	7.29	7.29	7.29	7.29	7.29	7.29	7.29	-9.00	-9.00	-9.00	-9.00	-9.00	-9.00	-9.00
26-Mar	-1.29	-1.29	-1.29	-1.29	-1.29	-1.29	-1.29	-10.29	-10.29	-10.29	-10.29	-10.29	-10.29	-10.29
27-Mar	-3.14	-3.14	-3.14	-3.14	-3.14	-3.14	-3.14	-13.43	-13.43	-13.43	-13.43	-13.43	-13.43	-13.43
28-Mar	-1.43	-1.43	-1.43	-1.43	-1.43	-1.43	-1.43	-14.86	-14.86	-14.86	-14.86	-14.86	-14.86	-14.86
29-Mar	-1.00	-1.00	-0.30	-0.29	-1.00	-1.00	-1.00	-15.86	-15.86	-15.16	-15.15	-15.86	-15.86	-15.86
30-Mar	-1.71	-1.71	-0.61	-0.60	-1.71	-1.71	-1.71	-17.57	-17.57	-15.77	-15.75	-17.57	-17.57	-17.57
31-Mar	-1.57	-1.57	-0.82	-0.81	-1.57	-1.57	-1.57	-19.14	-19.14	-16.59	-16.56	-19.14	-19.14	-19.14
01-Apr	-1.00	-0.82	-0.91	-0.90	-0.82	-1.00	-1.00	-20.14	-19.96	-17.50	-17.45	-19.96	-20.14	-20.14
02-Apr	-0.86	-0.92	-0.98	-0.96	-0.92	-0.86	-0.86	-21.00	-20.88	-18.47	-18.42	-20.88	-21.00	-21.00
03-Apr	0.00	-0.75	-0.82	-0.81	-0.75	0.00	0.00	-21.00	-21.63	-19.30	-19.23	-21.64	-21.00	-21.00
04-Apr	-0.86	-0.87	-0.92	-0.90	-0.88	-0.86	-0.86	-21.86	-22.50	-20.21	-20.13	-22.52	-21.86	-21.86
05-Apr	-1.57	-0.54	-0.68	-0.66	-0.53	-1.57	-1.57	-23.43	-23.04	-20.89	-20.79	-23.05	-23.43	-23.43
06-Apr	-2.14	-0.86	-0.97	-0.96	-0.86	-2.14	-2.14	-25.57	-23.89	-21.86	-21.75	-23.90	-25.57	-25.57
07-Apr	-2.43	-0.94	-1.07	-1.05	-0.94	-1.33	-0.94	-28.00	-24.84	-22.92	-22.80	-24.85	-26.90	-26.51
08-Apr	-2.43	-1.11	-1.23	-1.21	-1.12	-1.46	-1.11	-30.43	-25.95	-24.15	-24.01	-25.97	-28.36	-27.63
09-Apr	-1.71	-1.05	-1.19	-1.18	-1.05	-1.40	-1.05	-32.14	-27.00	-25.34	-25.19	-27.02	-29.76	-28.68
10-Apr	-1.57	-0.79	-0.95	-0.94	-0.78	-1.19	-0.79	-33.71	-27.79	-26.30	-26.13	-27.81	-30.96	-29.47
11-Apr	-3.00	-0.83	-0.99	-0.98	-0.83	-1.23	-0.83	-36.71	-28.63	-27.29	-27.10	-28.64	-32.19	-30.30
12-Apr	-2.43	-0.99	-1.20	-1.19	-0.99	-1.16	-0.99	-39.14	-29.62	-28.49	-28.29	-29.62	-33.35	-31.30
13-Apr	-1.43	-0.75	-0.97	-0.96	-0.73	-0.97	-0.75	-40.57	-30.37	-29.46	-29.25	-30.36	-34.32	-32.05
14-Apr	-1.14	-0.84	-1.03	-1.01	-0.84	-1.18	-0.84	-41.71	-31.21	-30.49	-30.26	-31.19	-35.51	-32.89
15-Apr	-1.00	-0.95	-1.11	-1.09	-0.95	-1.35	-0.95	-42.71	-32.16	-31.60	-31.35	-32.14	-36.86	-33.84
16-Apr	-1.00	-0.69	-0.87	-0.86	-0.69	-1.12	-0.69	-43.71	-32.85	-32.47	-32.21	-32.83	-37.98	-34.53
17-Apr	0.29	-0.73	-0.90	-0.89	-0.72	-1.09	-0.73	-43.43	-33.58	-33.38	-33.10	-33.55	-39.07	-35.26
18-Apr	-0.43	-0.54	-0.70	-0.68	-0.53	-1.07	-0.54	-43.86	-34.12	-34.07	-33.78	-34.08	-40.15	-35.79
19-Apr	-0.43	-0.75	-0.89	-0.88	-0.75	-1.18	-0.75	-44.29	-34.86	-34.97	-34.66	-34.83	-41.32	-36.54
20-Apr	-0.43	-0.27	-0.47	-0.46	-0.25	-0.65	-0.27	-44.71	-35.14	-35.44	-35.12	-35.08	-41.98	-36.81
21-Apr	-0.29	-0.71	-0.86	-0.84	-0.71	-1.05	-0.71	-45.00	-35.84	-36.30	-35.97	-35.79	-43.03	-37.52
22-Apr	0.00	-0.54	-0.70	-0.69	-0.53	-0.93	-0.54	-45.00	-36.38	-37.00	-36.65	-36.32	-43.96	-38.06
23-Apr	0.71	-0.30	-0.47	-0.46	-0.29	-0.69	-0.30	-44.29	-36.68	-37.47	-37.11	-36.61	-44.65	-38.36
24-Apr	-0.43	-0.19	-0.35	-0.34	-0.18	-0.63	-0.19	-44.71	-36.88	-37.82	-37.45	-36.79	-45.28	-38.55
25-Apr	0.00	-0.32	-0.49	-0.48	-0.32	-0.56	-0.32	-44.71	-37.20	-38.31	-37.93	-37.11	-45.84	-38.88
26-Apr	0.00	-0.69	-0.79	-0.77	-0.70	-1.03	-0.69	-44.71	-37.89	-39.10	-38.70	-37.81	-46.87	-39.57
27-Apr	-4.29	-0.12	-0.27	-0.26	-0.10	-0.45	-0.12	-49.00	-38.01	-39.37	-38.97	-37.91	-47.31	-39.68
28-Apr	-0.29	-0.78	-0.99	-0.98	-0.77	-0.39	-0.78	-49.29	-38.78	-40.36	-39.94	-38.68	-47.70	-40.46
29-Apr	-0.71	-0.30	-0.42	-0.41	-0.29	-0.47	-0.30	-50.00	-39.08	-40.78	-40.35	-38.97	-48.18	-40.76
30-Apr	-0.29	-0.50	-0.60	-0.58	-0.51	-0.64	-0.50	-50.29	-39.58	-41.37	-40.94	-39.48	-48.82	-41.26
01-May	-0.14	-0.50	-0.58	-0.57	-0.52	-0.72	-0.50	-50.43	-40.09	-41.95	-41.50	-40.00	-49.54	-41.76
02-May	-4.86	-0.36	-0.42	-0.41	-0.37	-0.54	-0.36	-55.29	-40.44	-42.37	-41.91	-40.37	-50.08	-42.12
03-May	-4.86	-1.01	-1.12	-1.11	-1.03	-0.47	-1.01	-60.14	-41.46	-43.49	-43.03	-41.40	-50.56	-43.14
04-May	-0.29	-0.85	-0.98	-0.97	-0.86	-0.27	-0.85	-60.43	-42.31	-44.46	-43.99	-42.26	-50.83	-43.99
05-May	1.14	-0.36	-0.41	-0.40	-0.37	-0.44	-0.36	-59.29	-42.67	-44.87	-44.39	-42.64	-51.27	-44.35
06-May	1.14	0.36	0.26	0.27	0.37	0.10	0.36	-58.14	-42.32	-44.61	-44.13	-42.27	-51.17	-43.99
07-May	1.14	0.36	0.30	0.31	0.37	0.18	0.36	-57.00	-41.96	-44.31	-43.82	-41.90	-50.99	-43.63
08-May	0.86	0.53	0.49	0.49	0.53	0.40	0.53	-56.14	-41.43	-43.81	-43.33	-41.37	-50.59	-43.11
09-May	5.71	0.49	0.48	0.48	0.49	0.45	0.49	-50.43	-40.94	-43.34	-42.85	-40.88	-50.14	-42.62
10-May	5.29	0.98	1.06	1.06	0.97	0.20	0.98	-45.14	-39.96	-42.28	-41.79	-39.91	-49.94	-41.64
11-May	5.14	0.99	1.10	1.10	0.99	0.27	0.99	-40.00	-38.97	-41.17	-40.69	-38.92	-49.67	-40.64
12-May	-0.29	1.19	1.30	1.29	1.19	0.53	1.19	-40.29	-37.78	-39.88	-39.40	-37.73	-49.15	-39.45
13-May	0.00	0.44	0.50	0.50	0.43	0.62	0.44	-40.29	-37.34	-39.37	-38.90	-37.31	-48.52	-39.02
14-May	-0.57	0.73	0.79	0.78	0.72	0.93	0.73	-40.86	-36.61	-38.59	-38.12	-36.59	-47.59	-38.29
15-May	0.43	0.66	0.74	0.73	0.66	1.01	0.66	-40.43	-35.95	-37.85	-37.39	-35.93	-46.59	-37.63
16-May	-0.14	1.18	1.24	1.23	1.19	1.45	1.18	-40.57	-34.77	-36.61	-36.17	-34.74	-45.13	-36.45
17-May	-0.14	0.89	0.98	0.97	0.88	1.31	0.89	-40.71	-33.88	-35.63	-35.19	-33.86	-43.82	-35.56
18-May	0.14	1.17	1.23	1.22	1.17	1.60	1.17	-40.57	-32.71	-34.40	-33.97	-32.69	-42.22	-34.39
19-May	0.14	1.34	1.41	1.39	1.35	1.79	1.34	-40.43	-31.37	-32.99	-32.58	-31.34	-40.43	-33.05

Figure 3A and B indicate the validation results by using different COVID-19 datasets. While only the result of cITS in Osaka doesn’t seem to estimate the counterfactual values well, the tendency is basically the same as in Tokyo (i.e., Fig. 2): compared to the counterfactual predictions made by our method, the conventional methods tend to underestimate the degree of reduction in HMI. The detailed values of the observed and estimated HMIs in the Additional files.

## Discussion

ITS analysis is frequently used to quantify the effects of health interventions on health outcomes at the population level. The most popular formulation of the ITS analysis is the (Gauss–Markov type) linear regression model, Eq. (1) [1, 6, 23]. One of the key assumptions is that the residuals are independent and not correlated. However, this assumption is often violated in time series data. By incorporating dependencies between different time points, the ARIMA model is a possible solution to this problem. In addition, the timing of the intervention is assumed to be clear; however, the actual time at which the effect begins is not always clear in practice. For example, as we described in “Application data analysis” section, the SoE itself was issued on a certain date, *T*_0_, but the actual influence was effective long before the issue of the SoE and distributed over time (i.e., [*T*_0_−*l*_1_,*T*_0_+*l*_2_]), as the media, such as TV, announced its issuance in advance. Thus, the actual timing of the SoE is unclear. To address these issues, we proposed the ARIMA ITS model with the distributed lagged functional terms *ARIMAITS-DL*. The lagged functional term is tailored to represent how the intervention effect is distributed before and after the nominal timing of the intervention (i.e., the date of the SoE). In addition to the distributed effect, another practical feature of our new model is that we can naturally model the cumulative effect with decaying parameter *δ*, which is explained in detail in “Detailed explanation on distributed lag functional terms” section. One possible difficulty highlighted by this abundance of choice (the orders (*p*,*d*,*q*), the distribution function *f*() with (*l*_1_,*l*_2_), and the restriction form for *w*_*k*_s) is how to choose the best combination. Another possible modeling techniques for the unclear timing of intervention would be the combination of fuzzy set theory and time series models, which have been extensively studied in the field of information science such as Chen (1996) and Singh (2021) [24, 25]. In addition, the fuzzy set theory has been frequently used for analyzing the COVID-19 data [26, 27]. These papers point the way to our next research directions.

In “Application data analysis” section, we used AIC to select the orders (*p*,*d*,*q*), and *f*(), and (*l*_1_,*l*_2_) were varied for the sensitivity check. However, a priori arguments and expert opinions may be helpful for this choice. A previous discussion concluded that the choice of the distributed lag terms should balance between sufficient complexity to capture detail and sufficient simplicity for interpretability from epidemiological or medical perspectives [28]. As we have no consensus on what is an “optimal” ARIMA model, sensitivity analyses and regression diagnoses such as residual analysis are particularly important to assess the robustness of the key conclusions. The R code, “fuzzyARIMAITS”, for the proposed method are provided in a GitHub repository (https://github.com/kingqwert/R/blob/master/ARIMAITS_DS/fuzzyARIMAITS.R) and will be hosted on the CRAN repository (https://www.r-project.org/) shortly, allowing others to apply our method easily.

Real data were examined with a detailed explanation of the analytical procedure to provide practical insights into the effect of the SoE on human mobility in Japan. We used four models consisting of two distributed lag functions (truncated normal and uniform distribution) and two restrictions on *w*_*k*_s (polynomial and MA) and confirmed that they exhibit almost the same effect for reducing human mobility in several COVID-19 datasets. Our results indicate that the SoE was successful in reducing the movement of people, at least during the study period (i.e., February 20 to May 19, 2020).

A limitation of this study is that our method was examined in only COVID-19 datasets while using four different settings, two comparison methods, and models with potentially different formulations associated with the distributed lag terms to check the sensitivity of the results. We encourage the re-evaluation of our approach using other datasets. Another limitation is that we assume that a stationary time series is available, which is the requirement of the AR model. The stationary assumption may be incompatible with time series data used in ITS analysis, where trends may change at certain points in time. However, in such a case, we can simply use ARIMA(0,0,0), and the idea of simultaneously using the ITS and distributed lag terms can still be valid. In this case, the model reduces to a simple form, such as Eq. (1), in which autocorrelation can be still modeled by including time as a covariate.

## Conclusion

The ITS model has been a powerful study design for evaluating health intervention impacts, and its use has been increasing. The most common formulation for ITS, Eq. (1), is not always adequate, especially when the timing of the intervention is unclear. Our model, *ARIMAITS-DL*, is a useful tool because it can account for such unclear intervention timing and distributed lag effect with autocorrelation and allows for flexible modeling of different types of impacts.

## Supplementary Information

**Additional file 1.** Observed and estimated human mobility index (HMI) in osaka and Ehime.

## Data Availability

The dataset generated during and/or analysed during the current study are available in https://www.google.com/covid19/mobility/?hl=en.
